# Magnetic targeting enhances the cutaneous wound healing effects of human mesenchymal stem cell-derived iron oxide exosomes

**DOI:** 10.1186/s12951-020-00670-x

**Published:** 2020-08-14

**Authors:** Xiuying Li, Ying Wang, Liyan Shi, Binxi Li, Jing Li, Zhenhong Wei, Huiying Lv, Liya Wu, Hao Zhang, Bai Yang, Xiaohua Xu, Jinlan Jiang

**Affiliations:** 1 Scientific Research Center, China-Japan Union Hospital of Jilin University, No. 126 Xiantai Street, Changchun, 130033 Jilin China; 2 State Key Laboratory of Supramolecular Structure and Materials, College of Chemistry, Jilin University, Changchun, Jilin China; 3 Department of Nephrology, China-Japan Union Hospital of Jilin University, No. 126 Xiantai Street, Changchun, 130033 Jilin China

**Keywords:** Exosome, Iron oxide nanoparticle, Mesenchymal stem cell, Cutaneous wound

## Abstract

Human mesenchymal stem cell (MSC)-derived exosomes (Exos) are a promising therapeutic agent for cell-free regenerative medicine. However, their poor organ-targeting ability and therapeutic efficacy have been found to critically limit their clinical applications. In the present study, we fabricated iron oxide nanoparticle (NP)-labeled exosomes (Exo + NPs) from NP-treated MSCs and evaluated their therapeutic efficacy in a clinically relevant model of skin injury. We found that the Exos could be readily internalized by human umbilical vein endothelial cells (HUVECs), and could significantly promote their proliferation, migration, and angiogenesis both in vitro and in vivo. Moreover, the protein expression of proliferative markers (Cyclin D1 and Cyclin A2), growth factors (VEGFA), and migration-related chemokines (CXCL12) was significantly upregulated after Exo treatment. Unlike the Exos prepared from untreated MSCs, the Exo + NPs contained NPs that acted as a magnet-guided navigation tool. The in vivo systemic injection of Exo + NPs with magnetic guidance significantly increased the number of Exo + NPs that accumulated at the injury site. Furthermore, these accumulated Exo + NPs significantly enhanced endothelial cell proliferation, migration, and angiogenic tubule formation in vivo; moreover, they reduced scar formation and increased CK19, PCNA, and collagen expression in vivo. Collectively, these findings confirm the development of therapeutically efficacious extracellular nanovesicles and demonstrate their feasibility in cutaneous wound repair.

## Introduction

Skin wound healing is a complex physiological process that involves inflammation, re-epithelialization, granulation, vascularization, and tissue remodeling [1]. Mesenchymal stem cell (MSC) therapy has been reported to be a promising therapeutic approach for wound healing [2], with an increasing number of studies demonstrating that MSCs elicit therapeutic effects neither by replacing damaged cells nor by implanting and differentiating [3–5]. Although considerable progress has been made in animal models, the clinical application of MSC-based therapies has been problematic [6], with the majority of the injected cells being washed away or displaying poor survival rates at the wound site. Moreover, MSC-based therapies must overcome significant regulatory barriers and require meticulous handling at all stages of harvesting, processing, and transplantation [7]. Because transplanted MSCs display limited viability in severe wound environments [8], it is necessary to develop novel strategies that can maximize the skin injury-repairing therapeutic effects of MSCs while avoiding the risks associated with their direct application.

Recent studies have shown that the majority of the therapeutic benefits of MSCs result from the paracrine actions of various cytokines and growth factors that affect the biological functions of skin cells, such as wound healing, scar formation, and photoaging [9–12]. MSC-derived exosomes (MSC-Exos), a type of lipid membrane-bound vesicle with a diameter of 30–150 nm, are a major component of this paracrine effect and an important regulator of intercellular communication [13, 14]. Indeed, an increasing number of studies have reported that MSC-Exos are highly promising cell therapy candidates for several diseases [15, 16]. Exos play a major role in intercellular communication by mediating the horizontal transfer of coding and noncoding RNAs and proteins to target cells, thereby altering their gene and protein expression to regulate their function [9]. Importantly, Exos have been reported to display functional properties similar to the MSCs from which they are derived without their significant adverse effects, such as vascular obstructive risk, malignant transformation, and immunogenicity, while also exhibiting strong cargo-loading and cargo-protective capacities. Thus, Exo-based therapies may be safer than the direct use of cells and offer a promising alternative to tissue regenerative applications [10–12, 17]. Recent studies have shown that many MSC-derived Exos can promote wound healing and accelerate skin regeneration by enhancing the proliferation and migration of related cells, promoting angiogenesis and re-epithelization, and regulating immune responses [18–20]. Although Exos may be a promising cell-free alternative to MSC therapy, MSC-Exo technology must be improved for clinical application. For instance, MSCs produce only a small number of Exos (1–4 μg of Exo proteins from 10^6^ cells per day); [21] therefore, a large number of MSCs must be cultured long term to produce sufficient MSC-Exos for clinical applications. However, late-passage MSCs display significantly reduced growth factor gene and protein expression [22], which would reduce the quantity of therapeutic growth factors and their mRNAs in the secreted Exos. Furthermore, because Exos display poor accumulation in the target organ after systemic administration in vivo, [23–26] modifications are necessary.

Superparamagnetic iron oxide nanoparticles (NPs) are a type of nanomaterial characterized by easy synthesis, superparamagnetism, high saturation magnetization, good biocompatibility, and low toxicity [27]. Nowadays, NPs are widely used in the biomedical field. For example, they can be designed and modified appropriately to serve as intelligent nanoprobes, which can respond to specific features in the tumor microenvironment to obtain real-time, high-resolution, cell-level and even molecular-level images of the tumor microenvironment [28–30]. As a result of their superparamagnetism, NPs can be magnetized up to their saturation magnetization by external magnetic guidance but display no residual magnetic interaction following removal of the magnetic guidance, conferring excellent dispersion and targeting capacities [31]. Moreover, when the magnetic force exceeds the linear blood flow rate, the NPs are retained in the required area. Owing to their advanced targeting capacities, biocompatibility, biodegradability, and low toxicity, NPs are considered a promising therapeutic tool [32]. Furthermore, recent studies have shown that Exos endowed with magnetic properties can be efficiently modulated by magnetic guidance, providing Exos with ideal targeting properties for tumor treatment [33, 34]. Therefore, Exos modified with targeting NPs may be a potential tool for disease treatment.

**Fig. 1:**
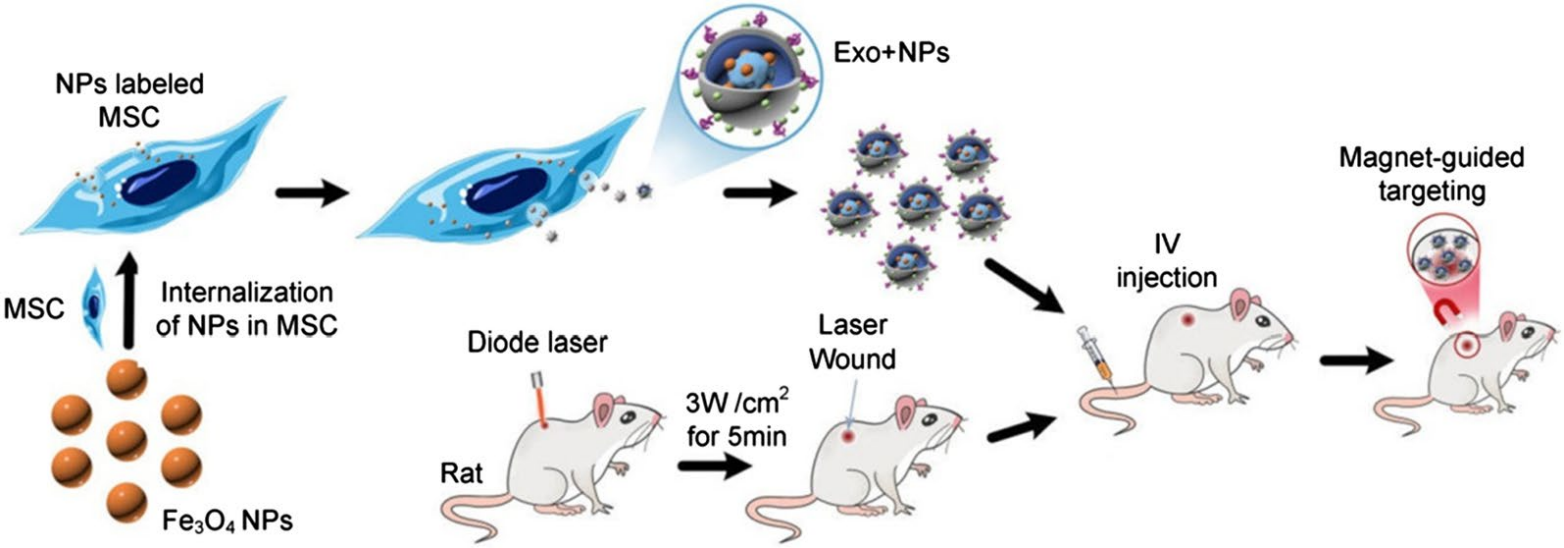
Schematic illustration of the preparation of NP-incorporated exosomes (Exo + NPs), from Fe_3_O_4_ NP-labeled MSCs, followed by magnet-guided in vivo targeting to the injured skin

In the present study, we extracted Exos from NP-loaded MSCs (MSC-Exo + NPs) to increase the in vivo targeting efficiency of MSC-Exos and verified their therapeutic cutaneous wound healing effect in vivo (Fig. 1). Moreover, to the best of our knowledge, we showed for the first time that external magnetic guidance enhances the targeted migration of Exo + NPs and increases the number of particles that home to the injured site, thereby facilitating skin wound healing by promoting collagen synthesis and angiogenesis.

## Results

### Fe_3_O_4_ NP characterization and cellular uptake

**Fig. 2:**
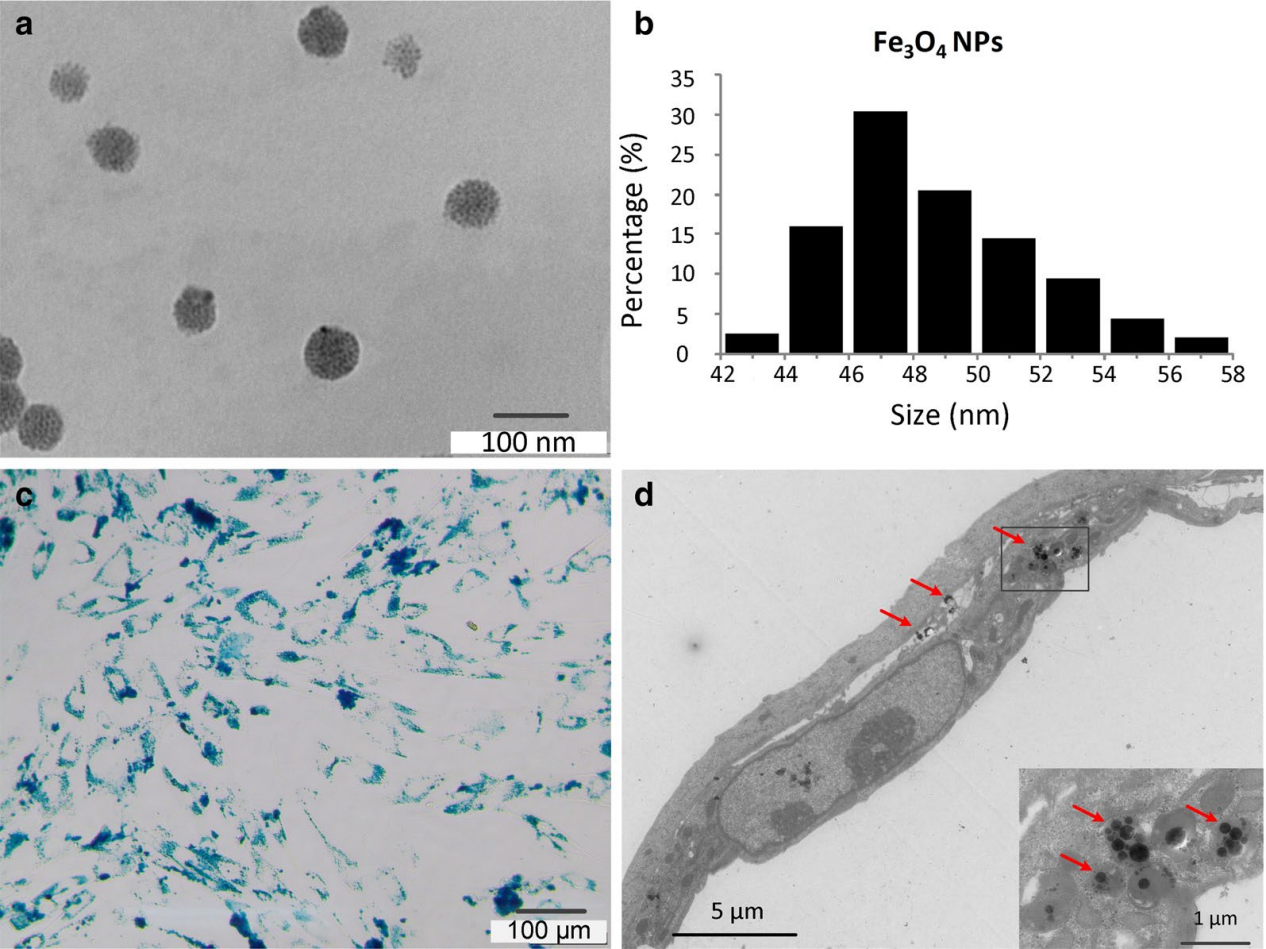
Fe_3_O_4_ nanoparticle (NP) characterization and internalization by mesenchymal stem cells (MSCs). **a** TEM images of Fe_3_O_4_ NPs. Scale bar = 100 nm. **b** Size distribution of Fe_3_O_4_ NPs. **c** MSCs were labeled with Fe_3_O_4_ NPs (50 μg/mL) for 16 h to determine the optimal labeling efficiency and stained using a Prussian blue iron staining kit. Scale bar = 100 µm. **c** TEM image of NPs (50 µg/mL) internalized by an MSC. Scale bar = 5 µm. Red arrows indicate NPs observed in the MSC cytoplasm

The morphology of the synthesized Fe_3_O_4_ NPs was analyzed using transmission electron microscopy (TEM). Figures 2a, b show TEM images displaying the morphology of magnetite NPs and the size distributions of Fe_3_O_4_ nanoparticles of < 60 nm, respectively. NPs (50 µg/mL) were efficiently internalized into MSCs. After Prussian blue staining, NPs were detected in MSCs as blue-stained dots (Fig. 2c). Moreover, the TEM images of MSCs revealed that the NPs aggregated in the cytoplasm, with close examination revealing that the aggregates were surrounded by membrane fragments in some images, indicating that they may have been in an endosomal compartment (Fig. 2d).

### Exo isolation and characterization

**Fig. 3:**
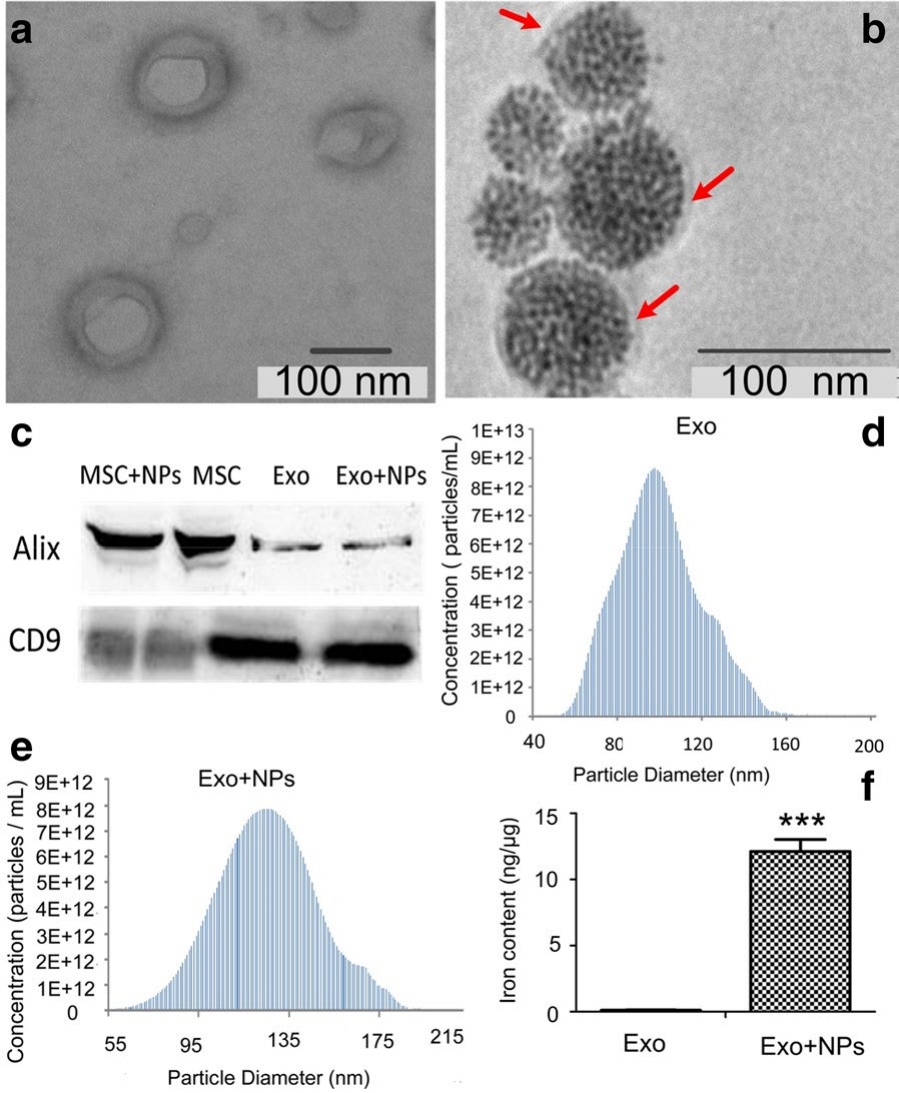
Mesenchymal stem cell -derived exosome (MSC-Exo) and MSC-Exo + NP characterization. Morphology of MSC-Exos (**a**) and MSC-Exos + NPs (**b**) observed by TEM. The red arrow indicates the membrane. **c** Western blot analysis of MSC-Exo and MSC-Exo + NP surface marker proteins (Alix and CD9). Size distribution of MSC-Exos (**d**) and MSC-Exo + NPs (**e**) determined by NP tracking analysis. **f** Iron content of MSC-Exos and MSC-Exo + NPs detected by ICP-OES analysis. ***P < 0.001 vs. MSC-Exos

Exos were isolated and purified from the supernatant of NP-loaded MSCs (Exo + NPs) and MSCs (Exos). TEM revealed that the Exo extracts contained round, cup-shaped vesicles of 50–150 nm (most were approximately 90 nm) with clear membrane structures and homogeneous size distribution (Fig. 3a). Besides, the TEM of Exo + NPs showed that the NPs were coated by membranes with a thickness of 7‒8 nm and that the NPs could be seen inside the Exos (Fig. 3b). Western blot analyses indicated that the Exos and the Exo + NPs expressed exosomal markers such as the Alix and CD9 proteins (Fig. 3c). Moreover, nanoparticle tracking analysis (NTA) showed that the size distribution peak of the Exos was 98.5 ± 1.4 nm (Fig. 3d), whereas that of the Exo + NPs was 116.7 ± 1.3 nm (Fig. 3e); this slight change in diameter may be related to the cargo inside the Exos. Collectively, these results demonstrated that the Exo + NPs maintained the integrity and properties of the Exos. Notably, the iron content of the Exo + NPs was markedly higher than that of the Exos (Fig. 3f), at approximately 12 ng of iron in 1 μg of Exo protein when normalized to the amount of protein.

### Exo uptake by human umbilical vein endothelial cells (HUVECs)

**Fig. 4:**
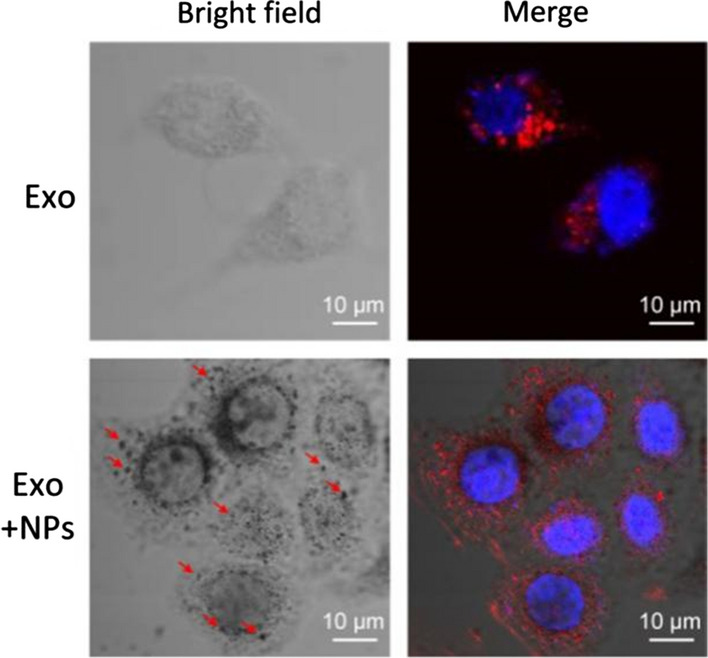
MSC-Exo and MSC-Exo + NP internalization. Confocal microscopy images showing Exos and Exo + NP incorporation in HUVECs. Blue indicates DAPI staining of the nucleus. Red indicates DiD-labeled Exos or Exo + NPs. Red arrows show cytoplasmic NPs in bright field. Scale bar = 10 μm

Next, we investigated whether Exos or Exo + NPs could enter HUVECs by performing an in vitro tracking experiment in which Exos or Exo + NPs were labeled with the red fluorescent lipophilic dye DiD and incubated with HUVECs for 18 h. Fluorescence confocal microscopy revealed that the DiD-labeled Exos were transferred into HUVECs, with Fe_3_O_4_ NPs visualized as black particles in the bright field (Fig. 4). The predominant localization of these Exos in the perinuclear region suggested that the Exos could enter HUVECs and thereby regulate their biological behavior.

### Exos promoted HUVEC proliferation, migration, and tube formation in vitro

**Fig. 5:**
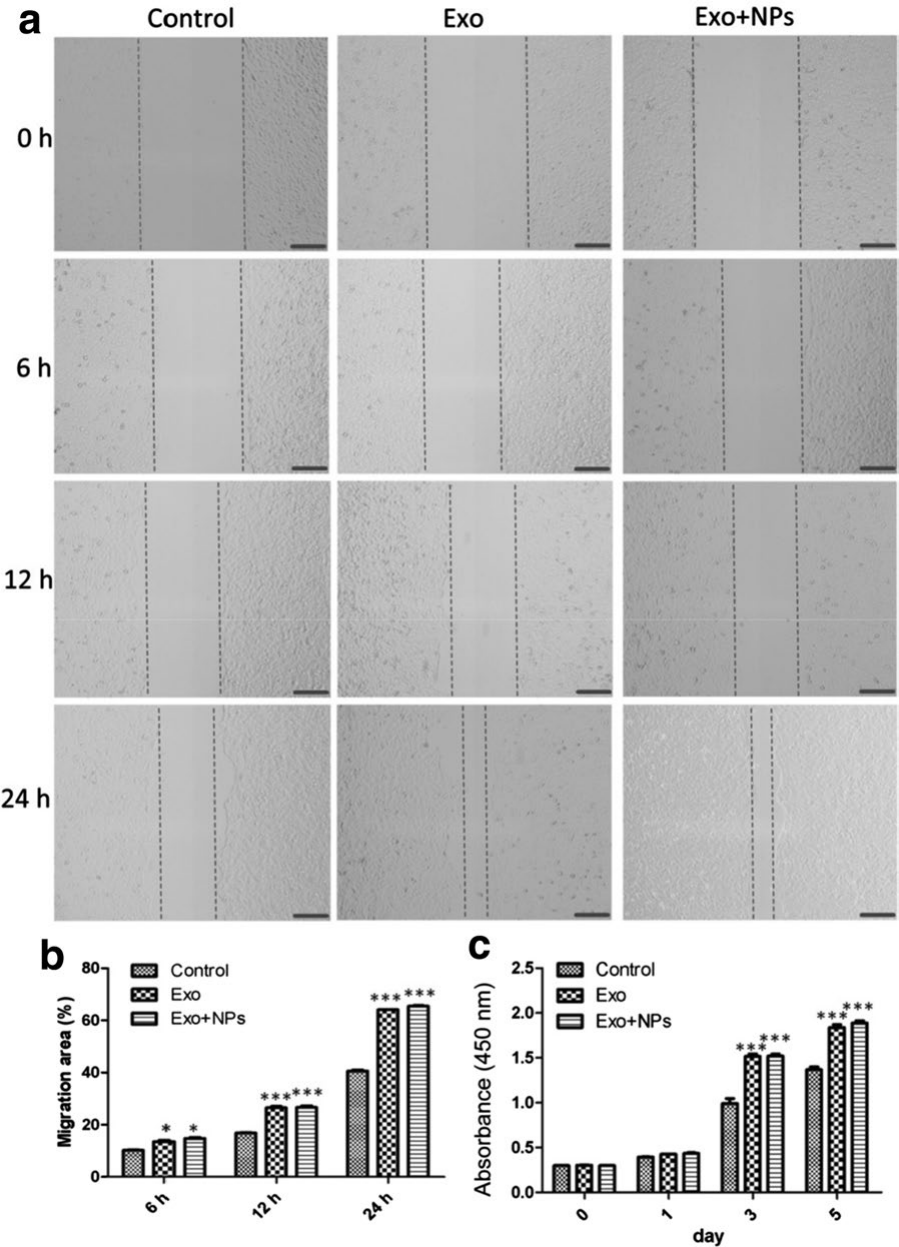
Effects of MSC-Exos or MSC-Exo + NPs on HUVEC proliferation and migration. Light microscopy images (**a**) and migration rates (**b**) of HUVECs into scratched monolayer areas following growth in fresh serum-free culture medium containing 50 μg/mL Exos or Exo + NPs for 6, 12, or 24 h. Scale bar = 200 μm. **c** Proliferation of HUVECs grown in medium containing 50 μg/mL Exos or Exo + NPs detected over 5 days using a cell counting kit. *P < 0.05, and ***P < 0.001

**Fig. 6:**
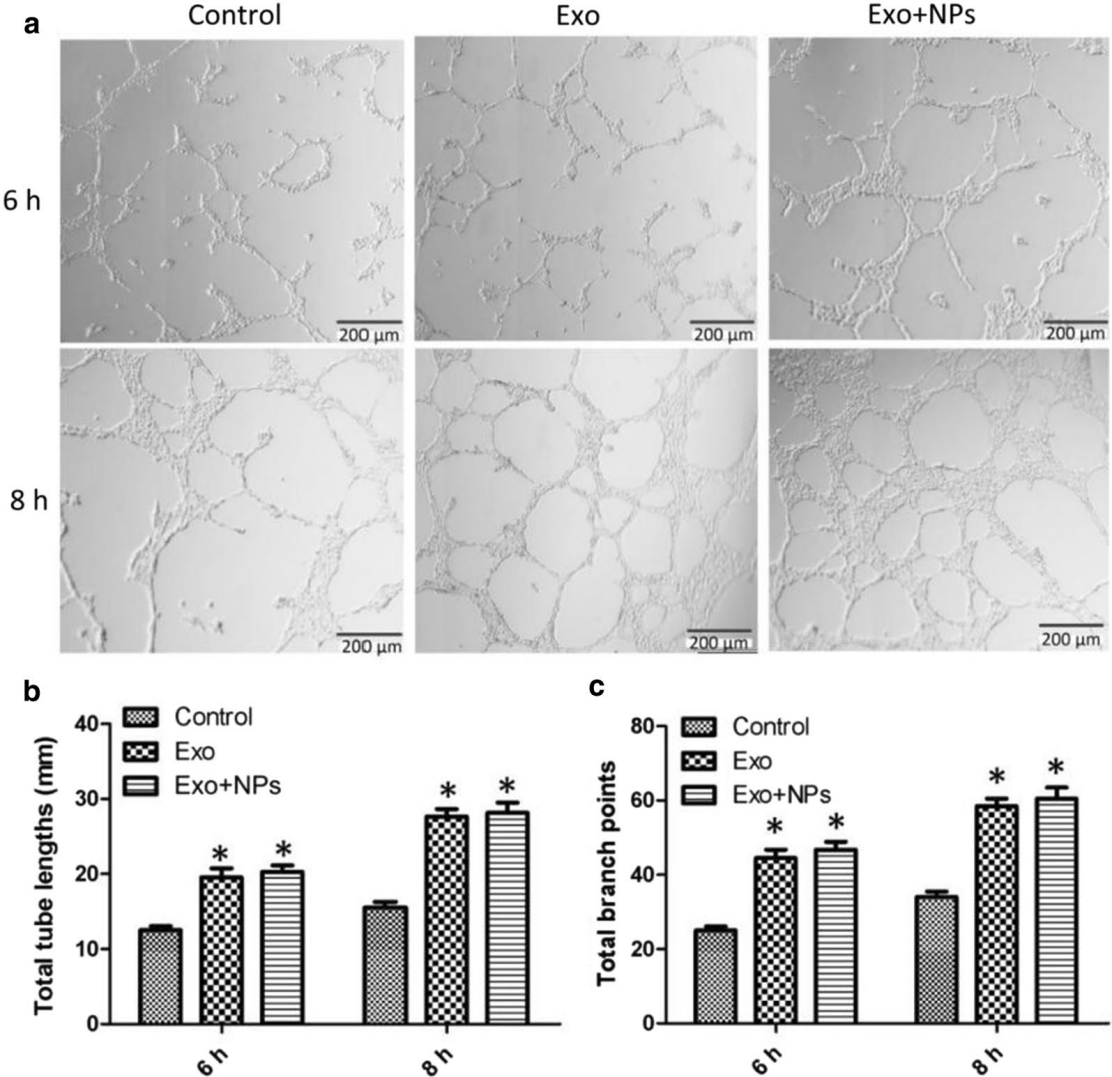
Pro-angiogenic effects of MSC-Exos or MSC-Exo + NPs on HUVECs. **a** HUVEC tube formation was studied by growing cells in Matrigel medium containing 50 μg/mL MSC-Exos or MSC-Exo + NPs. Scale bar = 200 μm. Quantitative analysis of the total tube length (**b**) and branch points (**c**) of HUVECs following growth in medium containing 50 μg/mL MSC-Exos or MSC-Exo + NPs for 6 or 8 h (*n* = 3 per group). *P < 0.05

To assess endothelial cell proliferation, migration, and tube formation capabilities, which are crucial during angiogenesis, we performed CCK8, scratch wound, and tube formation assays. MSC-Exos significantly enhanced the migration of HUVECs (Figs. 5a, b), whereas treatment with both Exos and Exo + NPs (50 μg/mL) markedly enhanced their proliferation capability (Fig. 5c). Moreover, improved tube formation was observed in the Exo and Exo + NP groups, as characterized by an increased number of tubes and complete tubular structures compared to those of the control group (Fig. 6a). In vitro tube formation assays were performed to evaluate the effect of Exos on HUVEC angiogenesis. As expected, the number of closed tubular structures increased by almost two-fold in the Exo and Exo + NP groups after 6 and 8 h of incubation, respectively, suggesting that Exos and Exo + NPs continuously promoted angiogenesis (Figs. 6b, c).

### Exos increased the S-phase fraction (SPF) and proliferation index (PIndex) of HUVECs

**Fig. 7:**
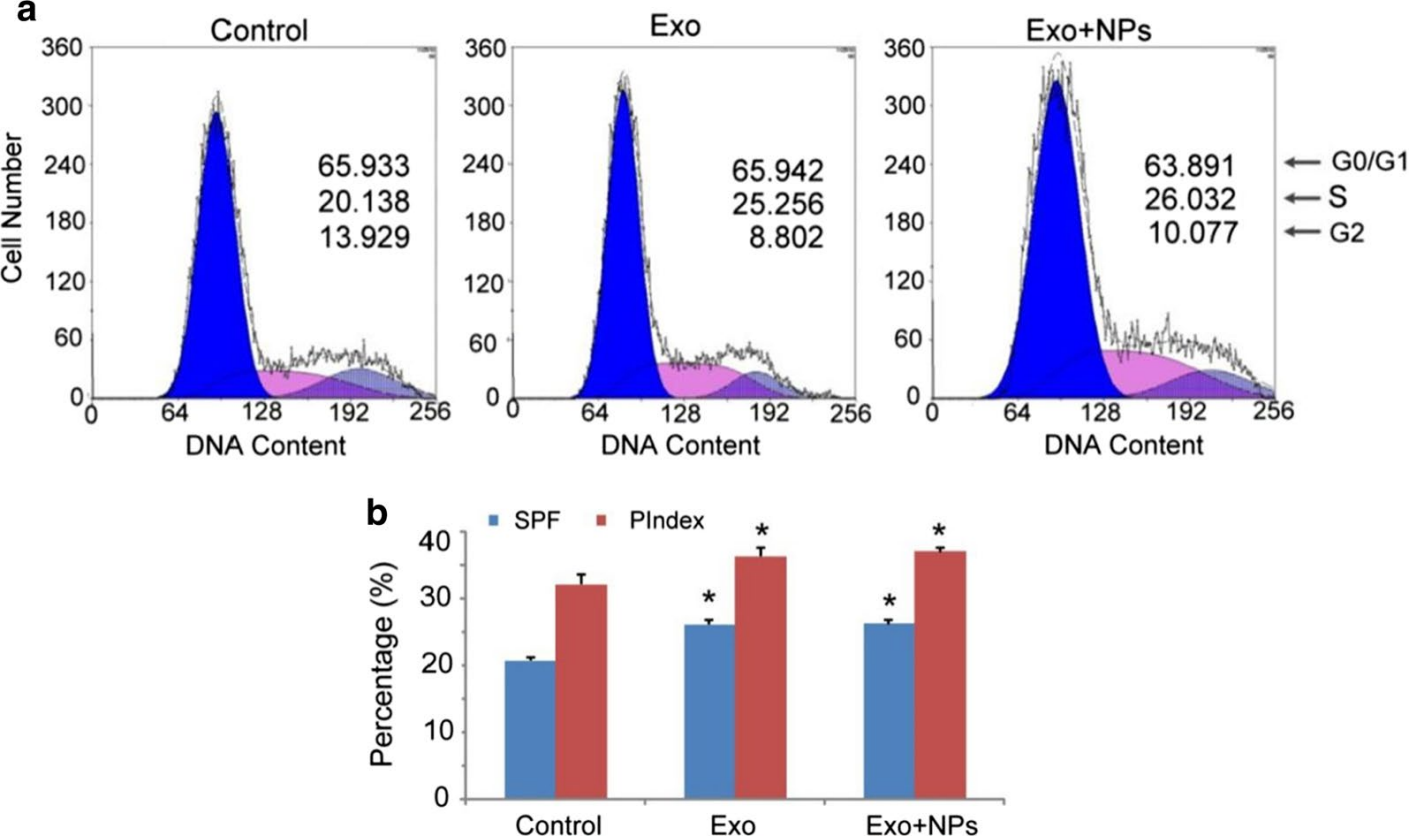
MSC-Exos increase HUVEC SPF and PIndex. HUVECs were cultured for 24 h with 50 µg/mL of MSC-Exos or MSC-Exo + NPs. The DNA content of the HUVECs was measured by propidium iodide staining using flow cytometry. **a** FACS plots representative of one of three experiments. **b** Averaged SPF and PIndex of three independent experiments. Bar = SD. *P < 0.05 vs. HUVEC group

Cell cycle analysis revealed that Exos and Exo + NPs increased the percentage of S-phase HUVECs compared to that of the controls (Fig. 7a). Moreover, Exo treatment significantly increased the SPF and PIndex of HUVECs compared to those of the controls (Fig. 7b), with no significant difference between the Exo and Exo + NP-treated groups. The results suggested that Exos and Exo + NPs could improve the proliferation capability of HUVECs by increasing the SPF and PIndex.

### Exos upregulated HUVEC proliferation-, migration-, and angiogenesis-related proteins

**Fig. 8:**
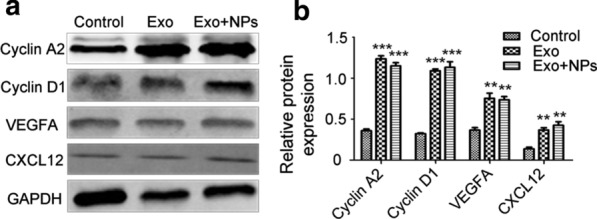
MSC-Exo treatment upregulated proteins associated with HUVEC proliferation, migration, and angiogenesis. Expression levels of cell cycle-, migration-, and angiogenesis- regulating proteins detected by western blot (**a**). GAPDH was used to normalize protein levels. **b** Quantification of Cyclin A2, Cyclin D1, VEGFA, and CXCL12 protein levels compared to the levels in the control group (mean ± SD; *n* = 3). **P < 0.01, ***P < 0.001 vs. control group

Next, we examined whether Exos could regulate protein expression associated with regenerative phenotypes. Western blot analyses revealed that Cyclin D1, Cyclin A2, VEGFA, and CXCL12 were all upregulated after Exo or Exo + NP treatment (Fig. 8a, b). These results showed that Exo and Exo + NPs increased HUVEC proliferation, migration, and angiogenesis capabilities through upregulation of the associated proteins.

### Exo transplantation promoted cutaneous wound healing in rats

**Fig. 9:**
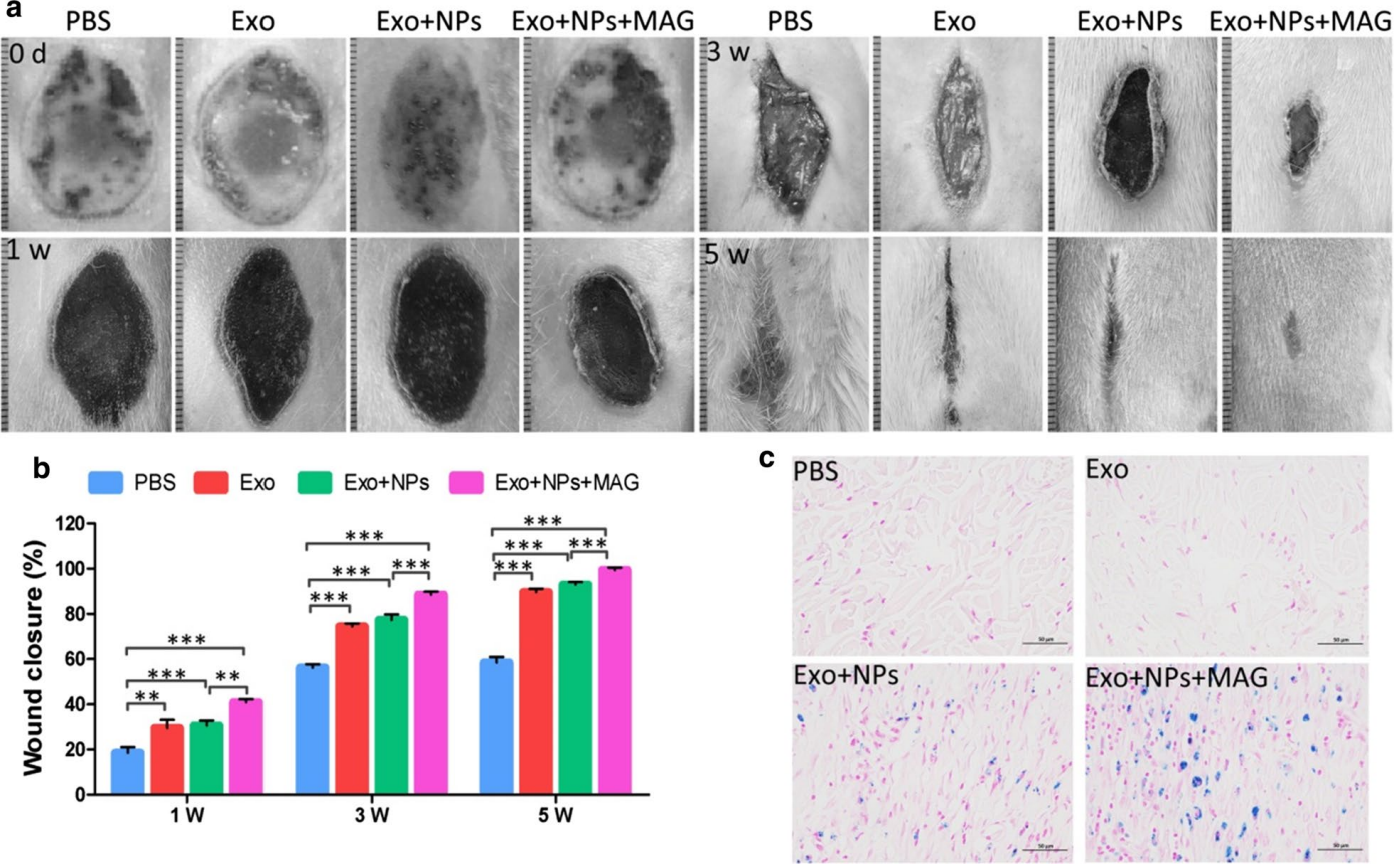
Macroscopic appearance of cutaneous wounds treated with PBS, Exos, Exo + NPs or Exo + NPs + MAG. **a** Gross view of wounds treated with PBS, Exos, Exo + NPs, or Exo + NPs + MAG after 0, 1, 3, and 5 weeks. **b** Quantitative analysis of wound closure at different time points post-wounding. *P < 0.05. After 24 h, **c** iron ion staining showed iron deposition (blue) in cutaneous wound tissue. Scale bar = 50 µm

We evaluated wound healing in four groups of rats injected with phosphate-buffered saline (PBS) (untreated group), Exos (Exo group), Exo + NPs (Exo + NP group), or Exo + NPs + MAG (Exo + NP with magnetic guidance group) via their tail veins. The Exo and Exo + NP groups had similar wound closures at weeks 3 and 5 post-wounding. Wound closure was greater in the rats treated with Exos or Exo + NPs than in the untreated groups at weeks 3 and 5 post-wounding. However, the animals in the Exo + NPs + MAG group showed the greatest wound closure (Figs. 9a, b), with their original wound area significantly smaller than that of the animals in the Exo- or Exo + NP-treated groups at weeks 3 and 5 post-wounding.

Combined with the properties of NPs, magnetic targeting for increasing the accumulation of NPs in cutaneous wounds potentially improve the delivery and retention of Exos in the wound microenvironment. The distribution of NP-labeled Exos was histologically evaluated by Prussian blue staining. The short-term distribution of NPs in rat cutaneous wound tissues was assessed after 24 h. As shown in Fig. 9c, the skin tissues of the magnetic field-exposed group exhibited higher iron density than did those of the Exo + NP-only group; most of the Exo + NPs moved toward the direction of the magnetic region. These findings suggested that external magnetic guidance promotes the homing of Exo + NPs to burn injury sites in vivo.

**Fig. 10:**
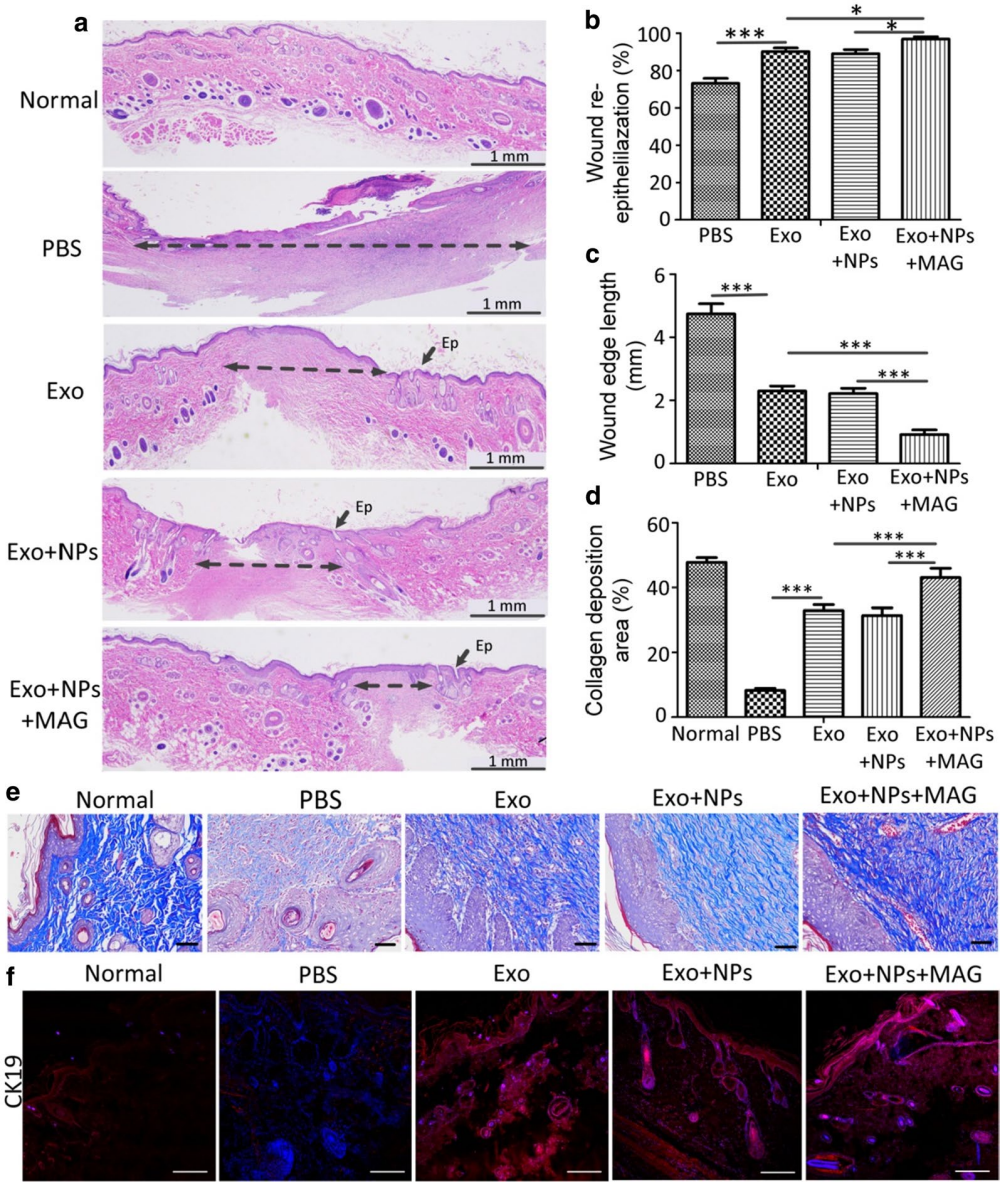
MSC-Exo + NPs with magnetic guidance accelerated the recovery of skin burn injury in rats. **a** H&E staining of wound sections following treatment with PBS, Exos, Exo + NPs, or Exo + NPs with magnetic guidance 5 weeks post-wounding. Double-headed arrows indicate scar edges. Scale bar = 1 mm; Ep, Epithelium. Effects of PBS, Exos, Exo + NPs, or Exo + NPs with magnetic guidance on wound re-epithelialization (**b**) and scar width (**c**) 5 weeks post-wounding (*n* = 6; *P < 0.05; ***P < 0.001). **d** Quantitative analysis of collagen in wound tissue 5 weeks after treatment (*n* = 5, ***P < 0.001). **e** Evaluation of collagen maturity by staining wounds with Masson’s trichrome following treatment with PBS, Exos, Exo + NPs, or Exo + NPs with magnetic guidance 5 weeks post-wounding. Scale bar = 100 μm. **f** Representative immunofluorescence images of CK19 expression showing re-epithelialization in the wound area. Scale bar = 100 μm

Reduced scar width and increased collagen maturity are indicators used to assess the degree of wound healing and regeneration. As shown in Figs. 10a–c, wound re-epithelialization was markedly enhanced, and wound edges were significantly narrower in the Exo + NPs + MAG group than in the Exo + NPs, Exo, and control groups at week 5 post-wounding. Moreover, greater and better-organized collagen deposition was observed in the wounds of rats in the Exo + NPs + MAG group than in those of the Exo + NPs, Exo, and control groups (Figs. 10d, e). To confirm the role of Exos in re-epithelialization, we detected the expression of CK19, an epithelial marker, using immunofluorescence staining. CK19 expression was markedly higher in the Exo, Exo + NPs, and Exo + NPs + MAG groups than in the control group 5 weeks post-wounding (Fig. 10f). Furthermore, the CK19-positive areas of the wounds in the Exo, Exo + NPs, and Exo + NPs + MAG groups had formed complete epidermal structures, unlike those of the control group. Collectively, these data indicated that Exo + NPs + MAG treatments significantly accelerated wound re-epithelialization and collagen deposition, and thus promoted wound healing. Moreover, increased Exo + NP retention at injury sites was associated with enhanced wound healing.

### Exo transplantation promoted angiogenesis and cellular proliferation in the wound sites of rats

**Fig. 11:**
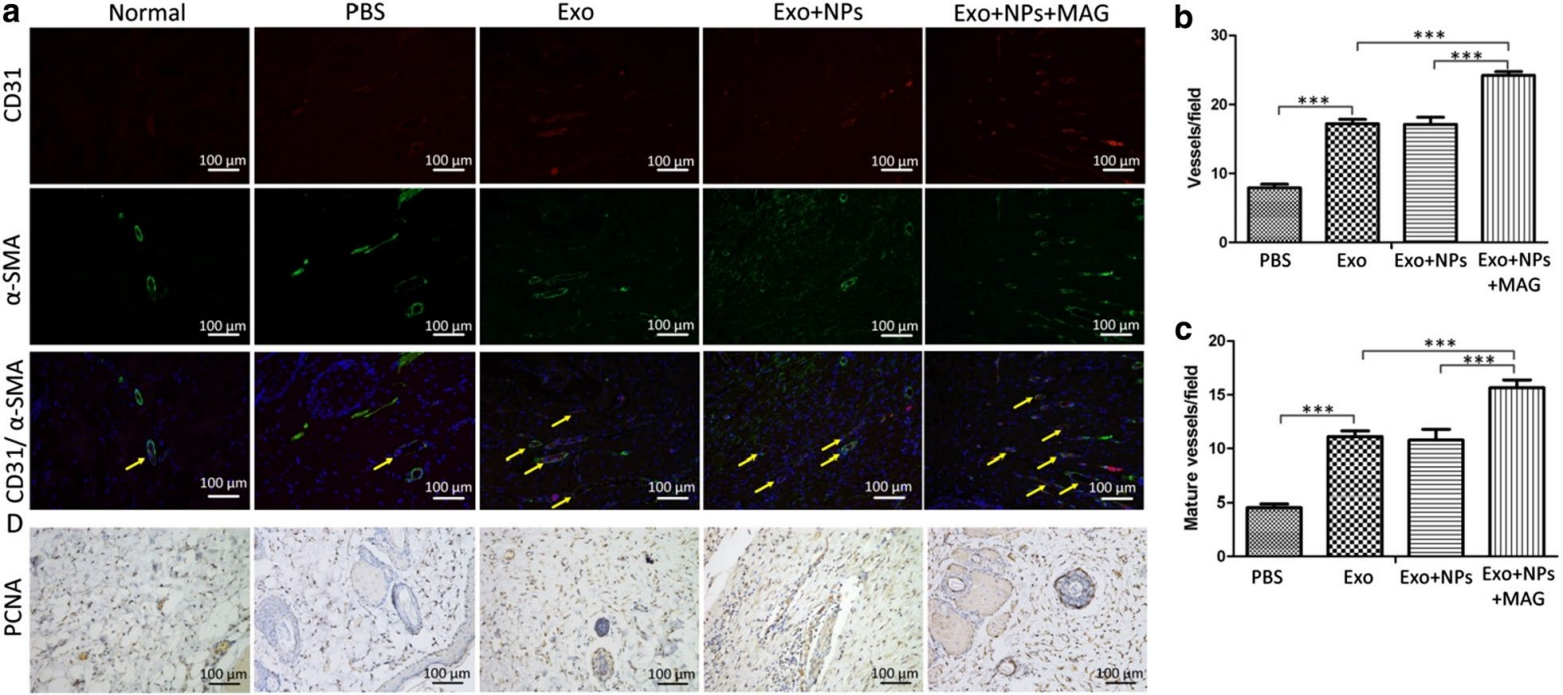
MSC-Exo + NPs with magnet-guided transplantation promoted the formation of new blood vessels in the wound sites of rats. **a** Immunofluorescence staining for CD31 and α-SMA in wounds after treatment with PBS, Exos, Exo + NPs, or Exo + NPs with magnetic guidance 5 weeks post-wounding. Scale bar = 100 μm. **b** Quantitative analysis of the number of total blood vessels by CD31 immunofluorescence staining. **c** Quantitative analysis of the number of mature blood vessels by CD31 and α-SMA double immunofluorescence staining in wounds. **d** Wounds were subjected to immunohistochemical staining for PCNA expression 5 weeks after treatment. Scale bar = 100 μm

The vascularization of newly formed tissues is an essential step in the wound healing process. In the present study, we identified newly formed and mature vessels at wound sites by CD31 staining or co-staining against CD31 and alpha-smooth muscle actin (α-SMA) (Fig. 11a), respectively, and then quantified the average vessel density and the number of mature vessels (Figs. 11b, c). We found that the number of newly formed and mature vessels increased during the healing process in all groups, with the Exo + NPs + MAG group displaying the greatest vessel density and number of mature vessels at week 5 and the Exo-treated group or Exo + NP-treated group having a higher vessel density and number of mature vessels than the control group. Furthermore, cellular proliferation was markedly enhanced in the groups treated with Exos, Exo + NPs, and Exo + NPs + MAG, as confirmed by the increased rate of PCNA + (Fig. 11d). Collectively, these results indicated that Exos, Exo + NPs and Exo + NPs + MAG promoted cellular proliferation and angiogenesis, which are the two primary wound healing processes, in vivo.

## Discussion

A significant number of clinical trials have demonstrated that MSCs can have beneficial therapeutic effects when used to heal cutaneous wounds, as well as in various diseases including myocardial infarction, bone defects, autoimmune diseases, and Crohn’s disease [35–38]. MSCs act via paracrine/endocrine mechanisms to trigger these regenerative processes, with these mechanisms also playing an important role in MSC-mediated repair [39]. Recent studies have demonstrated that Exos are important for the paracrine activity of MSCs [40], and it has been shown that the local injection of human MSC-derived Exos can accelerate cutaneous wound healing in vivo [19, 20]. However, it has been reported that intravenous injection is superior to local injection for wound healing, with the loss of Exos during local injection speculated to contribute to this difference. In addition, when Exos are injected directly into the wound, the wound is inevitably disturbed further, thus disrupting the wound healing process [35]. Therefore, in the present study, we used intravenous injection to study the effect of Exos on skin wound repair.

Previously, Pascucci et al. [41] showed that MSCs treated with paclitaxel incorporated the drug into Exos. Similarly, we also isolated NP-loaded Exos from NP-loaded MSCs. TEM images showed that labeled MSCs incorporated NPs by endocytic mechanisms, as already reported [42]. It has been demonstrated that NPs internalized by endocytosis often accumulate inside multivesicular bodies, which in turn may fuse with the plasma membrane thus releasing their cargo (Exos). [43, 44] Fe_3_O_4_ NPs likely undergo the same secretion process of the endosomal system. Exos effectively protect against tissue injury and exert a therapeutic effect in tissue repair [45, 46]. However, Exos have a limited ability to target injured tissues; therefore, it is necessary to enhance their targeting.

Because it has already been shown that the transplantation of NP-loaded MSCs promotes burn wound repair [47], the therapeutic effects of NP-loaded MSC-derived Exos for cutaneous wound repair expectedly offer good chances for clinical translation. As expected, the MSCs incorporated Fe_3_O_4_ NPs into Exos without affecting their characteristics in vitro. An increasing number of studies have reported that external magnetic fields can effectively control the localization of injected NPs in animals; [48, 49] for instance, magnetic guidance was shown to induce the accumulation of injected anti-cancer drug-attached NPs in the tumors of cancer patients [50, 51]. In the present study, external magnetic guidance promoted the homing of NP-loaded Exos to burn injury sites and improved wound repair in vivo.

Because angiogenesis involves the proliferation, migration, and angiogenic tubule formation of endothelial cells [52], we initially investigated the effects of Exos and Exo + NPs on the behavior of endothelial cells in vitro. Both Exos and Exo + NPs were able to integrate into endothelial cells (HUVECs) and significantly enhance their proliferation, migration, and angiogenic activity, thus confirming the pro-angiogenic properties of Exos. Additionally, Exo and Exo + NPs increased HUVEC proliferation, migration, and angiogenesis capability through upregulating proliferation-, migration-, and angiogenesis-related proteins, such as Cyclin D1, Cyclin A2, VEGFA, and CXCL12. Our in vitro results confirmed that both Exos and Exo + NPs could equally increase the proliferation, migration, and tube formation of HUVECs, while simultaneously upregulating the expression of related proteins. We further tested the targeting efficiency of Exo + NPs in a rat skin wound model. We observed a relatively significant accumulation of NPs at the injured skin site. Importantly, injected and magnet-guided Exo + NPs showed significantly enhanced accumulation at the site of injured skin, possibly due to the increased blood-circulation time of Exo + NPs and the external magnetic guidance. We further investigated whether the increased amount of Exo + NPs accumulated in injured skin exerts therapeutic effects. Encouragingly, our results also showed that Exo + NPs induced significant regenerative effects at the wound sites in a rat skin burn model, as defined by increased re-epithelialization and collagen deposition, more rapid wound closure, and reduced scar formation. Furthermore, we showed that Exo + NPs + MAG treatment markedly enhanced the number of total and mature blood vessels at the wound sites, with beneficial effects on blood vessel formation and cutaneous wound repair. The post-natal formation of new blood vessels occurs mainly through angiogenesis [53], which is essential for the survival, repair, and remodeling of injured tissues. Our results showed that Exo + NPs + MAG might improve angiogenesis at the wound site, increasing the blood vessel density and thereby accelerating the process of burn wound healing. Immunohistochemical staining for PCNA revealed that Exo + NPs + MAG could promote endothelial cell proliferation in vivo. We clearly observed that Exo + NPs + MAG treatment markedly increased re-epithelialization and collagen deposition at the wound site, whereas the new collagen fibrils did not exhibit periodic loss. Previous studies have demonstrated that higher concentrations of Exos are injected locally around the skin lesions and present a better therapeutic effect [18]. Our results also showed that magnetic targeting increases the number of Exo + NPs that accumulate in the injured area and that the therapeutic effect of magnetic field-guided Exo + NPs exceeds that of Exos or Exo + NPs without targeting. Of note, our results also indicated that Exo + NPs have similar tissue repair characteristics to MSCs; thus, Exo + NPs with magnetic field-guided targeting may serve as a promising candidate for treating skin wound healing and may overcome the barriers and risks associated with stem cell transplantation therapy.

Poor wound healing is often associated with abnormal blood supply to the wound bed. In contrast, studies have shown that Exos isolated from various cell types can promote angiogenesis and neovascularization [18, 19, 54]. In the present study, we found that magnetic targeting enhanced the cutaneous wound healing effects of Exo + NPs through significantly increasing the number of closed tubular structures in vitro and increasing the number of newly formed and mature blood vessels in vivo. These results indicated that Exo + NPs could improve the blood supply in wound beds and that the number of newly formed blood vessels increased as Exo + NPs accumulated in the wound. Thus, the rats in the Exo + NPs + MAG group displayed optimal wound healing effects.

## Conclusion

In the present study, we demonstrated the effective in vivo targeting ability of Exo + NPs and their feasibility for repairing cutaneous wounds. Similar to Exos, Exo + NPs can significantly promote the proliferation, migration, and angiogenesis of HUVECs in vitro and upregulate the expression of injured skin repair-related proteins, namely Cyclin A2, Cyclin D1, VEGFA, and CXCL12. In vivo magnetic guidance markedly enhanced the targeting efficacy of intravenously injected Exo + NPs toward the injured skin site and alleviated skin damage in a clinically relevant rat model. Our findings suggest that the application of Exo + NPs with magnetic guidance may be a promising therapeutic strategy for improving cutaneous wound healing, which may be applied to other types of tissue damages in patients. Thus, the noninvasive systemic application of Exo + NPs may represent a feasible therapeutic option for patients with cutaneous wounds. Moreover, this approach might be applied to treat various diseases including hindlimb ischemia and vascular injury in which tissue damage, angiogenesis, and tissue repair occur in this sequence.

## Materials and methods

### Synthesis of Fe_3_O_4_ NPs

Briefly, 5 mmol 1,2-hexadecanediol (90%, Sigma-Aldrich, St. Louis, Missouri, USA), 2 mmol iron acetylacetonate (Fe(acac)_3_, 99.9 + %, Sigma-Aldrich), 6 mmol oleic acid (OA, 90%, Sigma-Aldrich) and 6 mmol oleyamine (70%, Sigma-Aldrich) were mixed in 20 mL benzyl ether. Under nitrogen atmosphere, the mixture was heated to 200 ºC at a rate of 20 ºC min^−1^ for 30 min, and then refluxed for another 30 min at 265 ºC. Then, the solution was cooled to 23–25 ºC. The OA-stabilized Fe_3_O_4_ NPs were extracted and washed three times with ethanol before being dispersed in toluene. Under mechanical stirring and a nitrogen atmosphere at room temperature, 4.0 mL of 7.0 mg/mL OA-stabilized Fe_3_O_4_ NPs was injected into aqueous sodium dodecyl sulfate (SDS, 99%, Sigma-Aldrich) solution (2.8 mg/mL, 12.5 mL). After ultrasonic treatment, the emulsion was heated at 60 ºC to evaporate toluene. The SDS-capped Fe_3_O_4_ NPs were collected and stored at 4 °C in a refrigerator until use. Dynamic light scattering measurements were obtained using a Malvern Zetasizer Nano-ZS (Malvern Instruments, Worcestershire, UK).

### MSC preparation

MSCs were isolated from human umbilical cord tissue using our previously described method approved by the Ethics Committee of the China-Japan Union Hospital at Jilin University [47]. MSCs were cultured and expanded in α-minimum essential medium (MEM) supplemented with 10% fetal bovine serum (FBS; GIBCO, Clontech, Mountain View, CA, USA) at 37 °C and 5% CO_2_. MSCs from passages 3–6 were used in all experiments.

### Labeling of MSCs with Fe_3_O_4_ NPs

MSCs grown to 80% confluency were incubated with Fe_3_O_4_ NPs (50 µg/mL) for 16 h, washed three times with PBS, and stained with a Prussian blue iron staining kit (Solarbio, Beijing, China), according to the manufacturer’s instructions.

### MSC-derived Exo isolation and identification

MSCs (10^6^ cells) were incubated with 50 µg/mL of Fe_3_O_4_ NPs for 24 h and washed three times with PBS. Then, the culture medium was changed to α-MEM medium supplemented with 10% Exo-depleted FBS (SBI, Mountain View, CA, USA), and the cells were incubated for 48 h. The conditioned MSC medium (MSC-CM) was collected, centrifuged at 1500 rpm for 15 min to remove cells and cell debris, and filtered using a 0.22-μm syringe filter. The supernatant was passed through a 100-kDa molecular weight Amicon^®^ Ultra-15 Centrifugal Filter Device (Merck Millipore, Darmstadt, Germany) and concentrated. Exos were isolated from the MSC-CM using an exoEasy Maxi kit (Qiagen, Frankfurt, Germany) according to the manufacturer’s instructions. Briefly, the filtered MSC-CM was mixed at a 1:1 ratio with 2 × binding buffer (XBP) and added to an exoEasy membrane affinity column to allow the Exos to bind to the membrane. After centrifugation, the flow-through was discarded, and wash buffer (XWP) was added to the column to wash away nonspecifically retained material. After further centrifugation, the flow-through was discarded, and Exos were eluted by adding elution buffer to the spin column, with the eluate collected by centrifugation.

Exo morphologies were observed by 100 kV TEM, with size, concentration, and particle size distribution identified using NanoSight LM10 (Malvern, Worcestershire, UK) and NTA software version 3.0 (NanoSight, Malvern, Worcestershire, UK).

### Exo iron determination

Exos or Exo + NPs were lysed in 0.5 mL concentrated hydrochloric acid and their iron content quantified using an inductively coupled plasma optical emission spectrometer (ICP-OES) with a Perkin-Elmer Optima 3300DV (Perkin-Elmer, Norwalk, CT, USA).

### Exo labeling and internalization assay

Exos were incubated with a 1,1′-dioctadecyl-3,3,3′,3′-tetramethylindodicarbocyanine (DiD) tracer (5 µM; Sigma-Aldrich) for 4 min, treated with 0.5% BSA/PBS to neutralize excess dye, and the labeled Exos were obtained by centrifugation to remove contaminating dye. For the internalization assay, HUVECs were seeded in a 35-mm confocal dish at 2 × 10^5^ cells/dish and treated with 50 μg/mL labeled Exos. After incubation for 18 h, the cells were washed twice with PBS, fixed in 4% paraformaldehyde for 10 min, and their nuclei were stained with 4, 6-diamino-2-phenyl indole (DAPI, Solarbio, Beijing, China) according to the manufacturer’s instructions. Cellular Exo uptake was observed using laser scanning confocal microscopy.

### Cell proliferation assay

Cell growth was determined using Cell Counting Kit-8 (CCK-8; Sigma, St. Louis, MO, USA) assays. Briefly, HUVECs were seeded in 96-well plates (2000 cells/well) and co-cultured with Exos, Exo + NPs (50 μg/mL), or an equal volume of PBS. Cell growth was analyzed 1, 3, and 5 days after Exo treatment by measuring the optical density (OD) at 450 nm using a microplate reader (Bio-Rad Laboratories Inc., Hercules, CA, USA). Data are representative of three independent experiments.

### Cell migration assay

The effects of Exos on HUVEC migration were evaluated using a scratch assay. Briefly, cells were seeded in the cell culture system using an ibidi culture insert (ibidi GmbH, Munich, Germany). To measure cell migration, the silicon inserts were removed after 24 h, the gaps created were washed, and each well was filled with fresh serum-free culture medium containing Exos or Exo + NPs (50 μg/mL). Images of the closing area were obtained after 0, 6, 12, and 24 h, and the migration area was measured using Image-Pro Plus 6.0 software (Media Cybernetics, Inc., Rockville, MD, USA) as follows:

$${\text{Migration area }}\left( \% \right) \, = \, \left( {A_{0} {-}A_{n} } \right) \, /A_{0} \times 100,$$

where *A*_*0*_ represents the initial wound area (*t* = 0 h), and *A*_*n*_ represents the residual wound area at the time of measuring (*t* = *n* h).

### Tube formation assay

In vitro, capillary-like structure formation was evaluated using a Matrigel Basement Membrane Matrix (BD Biosciences, San Jose, CA, USA). Briefly, HUVECs (4 × 10^4^ cells/well) were seeded with 50 μg/mL Exos or Exo + NPs in 48-well culture plates coated with 150 μL Matrigel and cultured at 37 °C with 5% CO_2_. Tube formation was quantified after 6 and 8 h using an inverted microscope. The number of total branch points and tubule lengths in five randomly chosen fields were examined using an inverted microscope. Results represent the mean ± SEM of three independent experiments.

### Cell cycle

After incubation with 50 μg/mL of Exos or Exo + NPs for 24 h, HUVECs were collected, washed twice with PBS, and fixed with 70% alcohol at 4 °C for more than 24 h. The cells were then stained with 50 µg/mL of propidium iodide (PI) and 50 µg/mL of RNase A (Beyotime Institute of Biotechnology, Jiangsu, China) at 23–25 °C in the dark for 30 min, filtered, and measured using a flow cytometer (FC500; Beckman Coulter Inc., Fullerton, CA, USA). All data were collected and analyzed using flow cytometer software (Beckman Coulter Inc.). The S-phase fraction (SPF) was calculated as follows: SPF = S/(G0/G1 + S + G2/M) × 100%.

The proliferation index (PIndex) was calculated as follows: [55]

$${\text{PIndex }} = \, \left( {{\text{S }} + {\text{ G2}}/{\text{M}}} \right) \, / \, \left( {{\text{G}}0/{\text{G1 }} + {\text{ S }} + {\text{ G2}}/{\text{M}}} \right) \times 100 \, \% .$$

### Western blotting

Cells or purified Exo samples were diluted 1:5 with protein loading buffer (6 ×) (Transgen Biotech, Beijing, China) and heated at 99 °C for 10 min. Protein extracts were separated on a 10% sodium dodecyl sulfate–polyacrylamide gel electrophoresis gel and transferred onto polyvinylidene difluoride membranes (Sigma Aldrich Chemie GmbH, Munich, Germany) at 100 V for 30–60 min. The membranes were blocked with 5% nonfat milk at 23–25 °C for 1 h, washed three times in TBST buffer for 10 min, and incubated with the following primary antibodies at 4 °C overnight: CD9 (1:1000; BioLegend, San Diego, CA, USA), Alix (1:500; Santa Cruz Biotechnology, Santa Cruz, CA, USA), Cyclin A2 (1:1000; Proteintech Group, Rosemont, IL, USA), Cyclin D1 (1:5000; Proteintech), VEGFA (1:1000; Proteintech), CXCL12 (1:500; Proteintech), and GAPDH (1:5000; Proteintech). GAPDH was used as a loading control. Western blots were probed with IRDye 800-conjugated goat anti-rabbit or anti-mouse secondary antibodies and blotted proteins detected using an Odyssey infrared imaging system (LI-COR Biosciences, Lincoln, NE, USA).

### Rat skin wound model and treatment

Rats were handled in strict accordance with the Guidelines for the Care and Use of Laboratory Animals of Jilin University. All animal experiments were approved by the Ethics Committee of Animal Experiments of Jilin University and were carried out according to internationally accepted animal care guidelines (EEC Directive of 1986; 86/609/EEC). Six-week-old male Wistar rats were randomly divided into four groups (*n* = 9 per group): PBS group (100 μL PBS), Exo group (100 μg Exos dispersed in 100 μL PBS), Exo + NPs group (100 μg Exo + NPs dispersed in 100 μL PBS), and Exo + NPs + MAG group (100 μg Exo + NPs dispersed in 100 μL PBS). A 1.2-T magnet was placed under the injury site in the Exo + NPs + MAG group for 30 min. As described previously [47], a full skin thickness burn was induced on the back of the rat (3 W cm ^−2^ for 5 min) using an 808-nm diode laser (LEO Photonics, Beijing, China). Exos were administered intravenously to the animals in the burn injury group. Photographs were acquired at weeks 0, 1, 3, and 5, and the wound area measured using Image J software. The wound-size reduction was calculated as follows:

$${\text{Wound}} - {\text{size reduction }}\left( \% \right) \, = \, \left( {A_{0} {-}A_{t} } \right) \, /A_{0} \times \, 100,$$

where *A*_*0*_ is the initial wound area, and *A*_*t*_ is the wound area 1, 3, or 5 weeks post-wounding.

### Histological analysis

Skin tissues excised from the wound sites were fixed with 4% paraformaldehyde, dehydrated using a graded alcohol series, embedded in paraffin, and cut into 4-μm-thick longitudinal sections. The sections were stained with H&E for the histological analysis of wound repair and Masson staining to evaluate collagen accumulation. The sections were stained with Prussian blue iron stain kit (Servicebio, Wuhan, China) according to manufacturer’s instructions. Immunohistochemical staining evaluated the expression of PCNA in skin tissues.

### Immunofluorescence analysis

CD31 and α-SMA were detected by immunofluorescence staining to study Exo-induced angiogenesis during wound healing. Briefly, skin tissue excised from the wound sites was fixed in 4% paraformaldehyde, dehydrated in 30% sucrose solution, embedded in OCT, and cut into 4-μm-thick sections perpendicular to the wound surface. The sections were blocked in 1% BSA for 30 min at 23–25 °C, incubated with rabbit anti-CD31 (1:100; Abcam, Cambridge, UK) and mouse anti-α-SMA (1:50; Abcam) antibodies overnight at 4 °C, stained with secondary Alexa-Fluor 594-conjugated goat anti-rabbit and Alexa-Fluor 488-conjugated goat anti-mouse secondary antibodies (Abcam, 1:200), and then counterstained with DAPI.  To evaluate the re-epitheliazation in the wound area, we detected the expression of CK19 using immunofluorescence staining. The sections were incubated with rabbit anti-CK19 (1:500; Servicebio, Wuhan, China) antibodies overnight at 4 °C, stained with cy3-conjugated anti-rabbit secondary antibody (1:500; Servicebio), and then counterstained with DAPI. Images were acquired using an Olympus IX81 microscope (Tokyo, Japan). Newly formed vessels were indicated by CD31-positive staining, whereas mature vessels were detected as CD31 and α-SMA double-positive vascular structures. The numbers of newly formed and mature vessels were counted in five random fields per section between wound edges using Image-Pro Plus 6.

### Statistical analysis

All data are expressed as the mean ± standard deviation (SD). Between-group differences were assessed by one-way analysis of variance (ANOVA) using SPSS software. P values < 0.05 were considered significant.

## Data Availability

The datasets used to support the findings of this study are included in the article.

## References

[1] Castilho RM, Squarize CH, Gutkind JS (2013). Exploiting PI3K/mTOR signaling to accelerate epithelial wound healing. Oral Dis.

[2] Khosrotehrani K (2013). Mesenchymal stem cell therapy in skin: why and what for?. Exp Dermatol.

[3] Nagubothu SR, Sugars RV, Tudzarovski N, Andren AT, Bottai M, Davies LC (2020). Mesenchymal stromal cells modulate tissue repair responses within the injured vocal fold. Laryngoscope.

[4] Navas A, Magana-Guerrero FS, Dominguez-Lopez A, Chavez-Garcia C, Partido G, Graue-Hernandez EO (2018). Anti-inflammatory and anti-fibrotic effects of human amniotic membrane mesenchymal stem cells and their potential in corneal repair. Stem Cells Transl Med.

[5] Di G, Du X, Qi X, Zhao X, Duan H, Li S (2017). mesenchymal stem cells promote diabetic corneal epithelial wound healing through TSG-6-dependent stem cell activation and macrophage switch. Invest Ophthalmol Visual Sci.

[6] Trounson A, McDonald C (2015). Stem cell therapies in clinical trials: progress and challenges. Cell Stem Cell.

[7] Duscher D, Barrera J, Wong VW, Maan ZN, Whittam AJ, Januszyk M (2016). Stem cells in wound healing: the future of regenerative medicine? A mini-review. Gerontology..

[8] Kim N, Cho SG (2015). New strategies for overcoming limitations of mesenchymal stem cell-based immune modulation. Int J Stem Cells.

[9] Wu Q, Ji FK, Wang JH, Nan H, Liu DL (2015). Stromal cell-derived factor 1 promoted migration of adipose-derived stem cells to the wounded area in traumatic rats. Biochem Biophys Res Commun.

[10] Hocking AM, Gibran NS (2010). Mesenchymal stem cells: paracrine signaling and differentiation during cutaneous wound repair. Exp Cell Res.

[11] Kim WS, Park BS, Sung JH (2009). The wound-healing and antioxidant effects of adipose-derived stem cells. Expert Opin Biol Ther.

[12] Park BS, Jang KA, Sung JH, Park JS, Kwon YH, Kim KJ (2008). Adipose-derived stem cells and their secretory factors as a promising therapy for skin aging. Dermatol Surg.

[13] Vizoso FJ, Eiro N, Cid S, Schneider J, Perez-Fernandez R (2017). Mesenchymal stem cell secretome: toward cell-free therapeutic strategies in regenerative medicine. Int J Mol Sci.

[14] Colombo M, Raposo G, Théry C (2014). Biogenesis, secretion, and intercellular interactions of exosomes and other extracellular vesicles. Annu Rev Cell Dev Biol.

[15] Park KS, Bandeira E, Shelke GV, Lasser C, Lotvall J (2019). Enhancement of therapeutic potential of mesenchymal stem cell-derived extracellular vesicles. Stem Cell Res Ther..

[16] Akyurekli C, Le Y, Richardson RB, Fergusson D, Tay J, Allan DS (2015). A systematic review of preclinical studies on the therapeutic potential of mesenchymal stromal cell-derived microvesicles. Stem Cell Rev Rep.

[17] Fischer UM, Harting MT, Jimenez F, Monzon-Posadas WO, Xue H, Savitz SI (2009). Pulmonary passage is a major obstacle for intravenous stem cell delivery: the pulmonary first-pass effect. Stem Cells Dev.

[18] Zhang J, Chen C, Hu B, Niu X, Liu X, Zhang G (2016). Exosomes derived from human endothelial progenitor cells accelerate cutaneous wound healing by promoting angiogenesis through Erk1/2 signaling. Int J Biol Sci.

[19] Zhang J, Guan J, Niu X, Hu G, Guo S, Li Q (2015). Exosomes released from human induced pluripotent stem cells-derived MSCs facilitate cutaneous wound healing by promoting collagen synthesis and angiogenesis. J Transl Med.

[20] Zhang B, Wang M, Gong A, Zhang X, Wu X, Zhu Y (2015). HucMSC-exosome mediated-Wnt4 signaling is required for cutaneous wound healing. Stem Cells.

[21] Katsuda T, Tsuchiya R, Kosaka N, Yoshioka Y, Takagaki K, Oki K (2013). Human adipose tissue-derived mesenchymal stem cells secrete functional neprilysin-bound exosomes. Sci Rep..

[22] Mastri M, Lin H, Lee T (2014). Enhancing the efficacy of mesenchymal stem cell therapy. World J Stem Cells.

[23] Wiklander OP, Nordin JZ, O’Loughlin A, Gustafsson Y, Corso G, Mager I (2015). Extracellular vesicle in vivo biodistribution is determined by cell source, route of administration and targeting. J Extracell Vesicles.

[24] Hwang DW, Choi H, Jang SC, Yoo MY, Park JY, Choi NE (2015). Noninvasive imaging of radiolabeled exosome-mimetic nanovesicle using (99 m)Tc-HMPAO. Sci Rep.

[25] Lai CP, Mardini O, Ericsson M, Prabhakar S, Maguire C, Chen JW (2014). Dynamic biodistribution of extracellular vesicles in vivo using a multimodal imaging reporter. ACS Nano.

[26] Alvarez-Erviti L, Seow Y, Yin H, Betts C, Lakhal S, Wood MJ (2011). Delivery of siRNA to the mouse brain by systemic injection of targeted exosomes. Nat Biotechnol.

[27] Hohnholt MC, Geppert M, Dringen R (2011). Treatment with iron oxide nanoparticles induces ferritin synthesis but not oxidative stress in oligodendroglial cells. Acta Biomater.

[28] Zeng J, Jing L, Hou Y, Jiao M, Qiao R, Jia Q (2014). Anchoring group effects of surface ligands on magnetic properties of Fe_3_O_4_ nanoparticles: towards high performance MRI contrast agents. Adv Mater.

[29] Gao Z, Hou Y, Zeng J, Chen L, Liu C, Yang W (2017). Tumor microenvironment-triggered aggregation of antiphagocytosis (99 m) Tc-labeled Fe(3) O(4) nanoprobes for enhanced tumor imaging in vivo. Adv Mater.

[30] Ma T, Hou Y, Zeng J, Liu C, Zhang P, Jing L (2018). Dual-ratiometric target-triggered fluorescent probe for simultaneous quantitative visualization of tumor microenvironment protease activity and pH in vivo. J Am Chem Soc.

[31] Nitin N, LaConte LE, Zurkiya O, Hu X, Bao G (2004). Functionalization and peptide-based delivery of magnetic nanoparticles as an intracellular MRI contrast agent. J Biol Inorgan Chem.

[32] Fan CH, Cheng YH, Ting CY, Ho YJ, Hsu PH, Liu HL (2016). Ultrasound/magnetic targeting with SPIO-DOX-microbubble complex for image-guided drug delivery in brain tumors. Theranostics.

[33] Qi H, Liu C, Long L, Ren Y, Zhang S, Chang X (2016). Blood exosomes endowed with magnetic and targeting properties for cancer therapy. ACS Nano.

[34] Jia G, Han Y, An Y, Ding Y, He C, Wang X (2018). NRP-1 targeted and cargo-loaded exosomes facilitate simultaneous imaging and therapy of glioma in vitro and in vivo. Biomaterials.

[35] Hu L, Wang J, Zhou X, Xiong Z, Zhao J, Yu R (2016). Exosomes derived from human adipose mensenchymal stem cells accelerates cutaneous wound healing via optimizing the characteristics of fibroblasts. Sci Rep..

[36] Pytlik R, Rentsch C, Soukup T, Novotny L, Rentsch B, Kanderova V (2017). Efficacy and safety of human mesenchymal stromal cells in healing of critical-size bone defects in immunodeficient rats. Physiol Res.

[37] Chen Y, Zhao Y, Chen W, Xie L, Zhao ZA, Yang J (2017). MicroRNA-133 overexpression promotes the therapeutic efficacy of mesenchymal stem cells on acute myocardial infarction. Stem Cell Res Ther..

[38] Barnhoorn MC, Wasser M, Roelofs H, Maljaars PWJ, Molendijk I, Bonsing BA (2019). Long-term evaluation of allogeneic bone marrow-derived mesenchymal stromal cell therapy for Crohn’s disease perianal fistulas. J Crohn’s Colitis..

[39] Gnecchi M, Zhang Z, Ni A, Dzau VJ (2008). Paracrine mechanisms in adult stem cell signaling and therapy. Circ Res.

[40] Liang X, Ding Y, Zhang Y, Tse HF, Lian Q (2014). Paracrine mechanisms of mesenchymal stem cell-based therapy: current status and perspectives. Cell Transplant.

[41] Pascucci L, Cocce V, Bonomi A, Ami D, Ceccarelli P, Ciusani E (2014). Paclitaxel is incorporated by mesenchymal stromal cells and released in exosomes that inhibit in vitro tumor growth: a new approach for drug delivery. J Control Release.

[42] Alam SR, Stirrat C, Richards J, Mirsadraee S, Semple SI, Tse G (2015). Vascular and plaque imaging with ultrasmall superparamagnetic particles of iron oxide. J Cardiovasc Magn Resonance.

[43] Keller S, Sanderson MP, Stoeck A, Altevogt P (2006). Exosomes: from biogenesis and secretion to biological function. Immunol Lett.

[44] Malatesta M, Giagnacovo M, Costanzo M, Conti B, Genta I, Dorati R (2012). Diaminobenzidine photoconversion is a suitable tool for tracking the intracellular location of fluorescently labelled nanoparticles at transmission electron microscopy. Eur J Histochem.

[45] Wu R, Huang C, Wu Q, Jia X, Liu M, Xue Z (2019). Exosomes secreted by urine-derived stem cells improve stress urinary incontinence by promoting repair of pubococcygeus muscle injury in rats. Stem Cell Res Ther..

[46] Tan CY, Lai RC, Wong W, Dan YY, Lim SK, Ho HK (2014). Mesenchymal stem cell-derived exosomes promote hepatic regeneration in drug-induced liver injury models. Stem Cell Res Ther..

[47] Li X, Xu Z, Bai J, Yang S, Zhao S, Zhang Y (2016). Umbilical cord tissue-derived mesenchymal stem cells induce T lymphocyte apoptosis and cell cycle arrest by expression of indoleamine 2, 3-dioxygenase. Stem Cells Int..

[48] Zhu LL, Zhang Z, Jiang HS, Chen H, Chen Y, Dai YT (2017). Superparamagnetic iron oxide nanoparticle targeting of adipose tissue-derived stem cells in diabetes-associated erectile dysfunction. Asian J Androl.

[49] Yanai A, Hafeli UO, Metcalfe AL, Soema P, Addo L, Gregory-Evans CY (2012). Focused magnetic stem cell targeting to the retina using superparamagnetic iron oxide nanoparticles. Cell Transplant.

[50] Wilson MW, Kerlan RK, Fidelman NA, Venook AP, LaBerge JM, Koda J (2004). Hepatocellular carcinoma: regional therapy with a magnetic targeted carrier bound to doxorubicin in a dual MR imaging/conventional angiography suite–initial experience with four patients. Radiology.

[51] Lubbe AS, Bergemann C, Riess H, Schriever F, Reichardt P, Possinger K (1996). Clinical experiences with magnetic drug targeting: a phase I study with 4′-epidoxorubicin in 14 patients with advanced solid tumors. Cancer Res.

[52] Yoo SY, Kwon SM (2013). Angiogenesis and its therapeutic opportunities. Mediators Inflamm.

[53] Bulgin D (2015). Therapeutic angiogenesis in ischemic tissues by growth factors and bone marrow mononuclear cells administration: biological foundation and clinical prospects. Curr Stem Cell Res Ther.

[54] Zhang W, Bai X, Zhao B, Li Y, Zhang Y, Li Z (2018). Cell-free therapy based on adipose tissue stem cell-derived exosomes promotes wound healing via the PI3K/Akt signaling pathway. Exp Cell Res.

[55] Zhang H, Qu S, Li S, Wang Y, Li Y, Wang Y (2013). Silencing SATB1 inhibits proliferation of human osteosarcoma U2OS cells. Mol Cell Biochem.

